# Risk Factors Associated with Acute Sarcopenia in Patients Hospitalized with COVID-19

**DOI:** 10.1155/2024/7857489

**Published:** 2024-03-12

**Authors:** D. M. S. Silva, T. A. Valadão, C. Caporosi, J. E. Aguilar-Nascimento, D. B. Dock-Nascimento

**Affiliations:** ^1^Graduate Program in Health Sciences, Federal University of Mato Grosso, Cuiabá, MT, Brazil; ^2^Postgraduate Program in Health Sciences, Federal University of Mato Grosso, Cuiabá, MT, Brazil; ^3^Santa Rosa Hospital, Cuiabá, MT, Brazil; ^4^University Center of Várzea Grande (UNIVAG) Medical School, Várzea Grande, MT, Brazil; ^5^Faculty of Nutrition of the Federal University of Mato Grosso (UFMT), Cuiabá, Brazil

## Abstract

**Background:**

The COVID-19 pandemic is an extraordinary global emergency. The pandemic has changed profoundly people's lifestyles. This resulted in reductions in physical activity and changes in dietary intakes that have the potential to accelerate sarcopenia.

**Objective:**

The aim of this study was to evaluate the risk factors associated with acute sarcopenia in patients hospitalized with COVID-19.

**Methods:**

This was a cross-sectional study conducted from January/2021 to March/2022 in a private hospital in Cuiabá/MT, central region of Brazil. The main variable was the prevalence of acute sarcopenia among adults hospitalized with COVID19. Patients were assessed for acute sarcopenia using the SARC-F ≥4 questionnaire (strength, assistance with walking, rise from a chair, climb stairs, and falls), grip strength (<20 kg (female) and <35 kg (male)), and calf circumference (<33 cm (female) and <34 cm (male)).

**Results:**

In all, 213 patients aged 57.4 ± 15.4 years, 63.8% male, were studied. Thirty-four (16.0%) patients were diagnosed with acute sarcopenia. Advanced age (older people) and the percentage of weight lost ≥3% before hospitalization were independent risk factors for acute sarcopenia in hospitalized patients with COVID-19.

**Conclusion:**

Acute sarcopenia was present in 16% of patients. Advanced age and percentage of weight lost ≥3% were independent risk factors for acute sarcopenia in patients hospitalized with COVID-19.

## 1. Introduction

The COVID-19 pandemic has changed profoundly people's lifestyles. The changes associated with COVID-19 infection include acceleration of muscle mass loss and a decline in functional capacity, the primary features of sarcopenia [1]. In addition, COVID-19 is an infectious disease characterized by mild to severe symptoms and an increase in metabolism and inflammatory cytokine production that leads to proteolysis and consequent muscle wasting [2]. Furthermore, the virus uses the cellular receptor of angiotensin-converting enzyme 2 (ACE2) for the replication process [3, 4]. ACE2 receptors are present in a ubiquitinated form, which results in damage and destruction of tissues, including muscle [5–7]. In this scenario, patients with COVID-19 have severe myalgia, weakness, fatigue, and rapid weight loss [2, 3]. Some patients even lose seven kilograms in two weeks [2]. In this context, the patient may evolve with worsening respiratory function requiring hospitalization. Following this, the patient may experience malnutrition and sarcopenia secondary to COVID-19, which is termed acute sarcopenia [8–11]. Acute sarcopenia is acute muscle insufficiency, defined by declines in muscle function and/or quantity within six months, usually following a stressor event such as COVID-19 [11]. Sarcopenic patients have the worst prognosis and may evolve with adverse outcomes during hospitalization for COVID-19 [10, 12]. Advanced age, malnutrition, and inflammatory processes are potential triggers of acute sarcopenia [11, 13, 14]. Studies with patients hospitalized for SARS-CoV-2 have described evidence of severe muscle damage [15], and it is speculated that these patients are at increased risk of developing acute sarcopenia [2, 16]. The aim of this study was to evaluate the risk factors associated with acute sarcopenia in hospitalized patients with COVID-19.

## 2. Methods

A cross-sectional study was conducted including consecutive hospitalized patients (≥18 years old) with COVID-19 in a private hospital (Santa Rosa) in Cuiabá-MT (January/2021 and March/2022). All participants signed informed consent which had been approved by the local ethical review boards. This study was realized with the guidelines laid down in the Declaration of Helsinki. The local clinical research ethics committee (CAE 38075220.5.0000.5541) approved this study.

### 2.1. Inclusion and Exclusion Criteria

All patients hospitalized with COVID-19 who, within the first 48 hours, were breathing spontaneously or using a nasal catheter without alteration in the level of consciousness were eligible. Only patients diagnosed with COVID-19 by PCR for SARS-CoV-2 from nasopharyngeal swabs were included. The SARS-CoV-2 variants identified during data collection were alpha, gamma, delta, and omicron [17]. Patients with difficulty answering the questions, those whose data were lost, those who at some point refused to participate in the research, and those who, within 48 hours of data collection and admission, were transferred to the intensive care unit (ICU) were not included.

### 2.2. Investigated Variables and Data Collection

Our primary outcome was the prevalence of acute sarcopenia among patients hospitalized with COVID-19. All data were collected within the first 48 hours of admission. Demographic characteristics (age, sex), nutritional therapy (TN), serum C-reactive protein (CRP; mg/L) levels, length of stay (LOS), and death were recorded from the electronic medical record. The comorbidities, body weight (BW; kg), usual body weight (UBW; kg), unintentional weight loss (kg and %), days, and signs and symptoms of prehospitalization were determined from patient-reported medical histories.

### 2.3. Assessment of Acute Sarcopenia

The SARC-F questionnaire was applied, followed by the assessment of muscle strength (handgrip strength, HGS/kg) and muscle mass (MM) (calf circumference, CP/cm) [10]. Patients who were at risk for sarcopenia by SARC-F (score ≥4) [18] with reduced HGS and CP were classified with acute sarcopenia. HGS was determined on the dominant hand (hydraulic dynamometer, Jamar®) [10, 18]. HGS <20 kg (females) or <35 kg (males) was defined as low [19]. CP was measured on the dominant leg with an inelastic measuring tape. Low MM was defined as CP <33 cm (female) or <34 cm (male) [20]. All data were collected by a previously trained dietitian.

### 2.4. Statistical Analysis

The Kolmogorov–Smirnov test was used to determine the normality of continuous data. If normally distributed, the data were summarized as the mean (*M*) and standard deviation (±SD), and if the distribution was asymmetrical, the data were summarized as the median (*M*) and interquartile range (IQR). To compare continuous variables, student's *t*-test was used if the observations between the groups met the assumptions of normality and homogeneity of variances. Otherwise, the nonparametric Mann–Whitney test was used. The chi-square test was used to analyze the association between categorical variables and the prevalence of acute sarcopenia. The epidemiological association was evaluated by determining the odds ratio (OR) and respective 95% confidence interval (95% CI). The percentage of weight lost was categorized as ≥3%. The variables that were associated with *p*  <  0.20 in the univariate comparison were inserted into a multivariate logistic regression model (stepwise automatic model). Moreover, for the multivariate model, only categorical variables and those that did not establish collinearity were used. For all analyses, a significance limit of 5% (*p*  <  0.05) was established. The Statistical Package for the Social Sciences 20.0 program (SPSS Statistics; IBM, Armonk, NY, USA) was used for statistical analyses.

## 3. Results

### 3.1. Patient Characteristics

**Figure 1:**
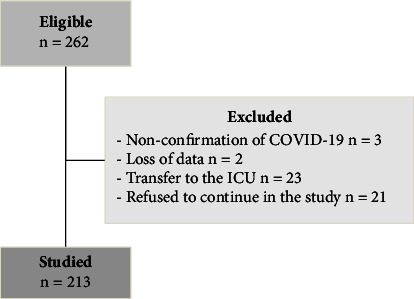
Flowchart of the study sample.

A total of 262 patients were eligible, of which 3 were excluded due to nonconfirmation of COVID-19, 2 due to loss of data, 23 due to transfer to the ICU, and 21 due to refusal to continue in the study (Figure 1). Thus, 213 patients hospitalized with COVID-19, with a mean (±SD) age of 57.4 ± 15.4 years, 136 (63.8%) of whom were male (Table 1), were studied. Patients reported a median time (IIQ) of 9.0 (7–11) days of symptoms before hospitalization, with a median LOS of 11 (5–19) days, and 16.4% (*n* = 35) evolved to death. The general patient characteristics are described in Table 1.

### 3.2. Prevalence of Acute Sarcopenia

The risk of acute sarcopenia was identified in 123 (42.3%) patients, 99 (46.5%) had low muscle strength, 48 (22.5%) had low MM, and 34 (16%) were diagnosed with acute sarcopenia. There was no difference between patients with and without acute sarcopenia in the results of PCR for C-reactive protein (*p*=0.474), symptom duration (*p*=0.607), or length of hospitalization (*p*=0.943) (Table 2).

### 3.3. Univariate and Multivariate Analyses of Factors Association with Acute Sarcopenia

The univariate analysis (Table 3) showed that gender (*p*=0.292), arterial hypertension (*p*=0.893), diabetes mellitus (*p*=0.703), presence of signs and symptoms (*p* > 0.05) except anorexia (*p*=0.027), and death (*p*=0.835) showed no association with acute sarcopenia. On the other hand, nonsarcopenic patients were more obese than sarcopenic patients (*p*=0.005). After adjustments, in multivariate analysis, advanced age (elderly, defined as ≥60 years) and a percentage of weight lost unintentionally ≥3% were risk factors independently associated with acute sarcopenia. However, a percentage of weight lost ≥3% showed almost twice the risk (Exp (B) = 6.93) for acute sarcopenia in patients hospitalized for COVID-19 (Table 4).

## 4. Discussion

Our results showed that 16% of patients with COVID-19 had already been hospitalized with acute sarcopenia. Furthermore, advanced age and ≥3% unintentional weight loss before admission were independent risk factors for acute sarcopenia among patients. Although 43.2% of the sample was composed of elderly people, in whom the probability of sarcopenia was increased more than 4-fold in the univariate analysis, in the multivariate analysis, ≥3% unintentional weight loss showed almost twice the risk for acute sarcopenia when compared to old age. In this scenario, it should be noted that primary sarcopenia is associated with a decline in muscle mass and strength in the fourth decade of life and is one of the primary contributors to disability in the elderly [10, 21]. In addition, during the COVID-19 pandemic [10, 21], the need for confinement contributed greatly to the reduction of physical activity and an increase in sedentary behavior, along with less healthy eating [22, 23]. These factors are associated with loss of muscle mass and worsening of muscle quality [24, 25]. All this, combined with SARS-CoV-2 infection, may have contributed to a greater loss of weight, function, and muscle mass, especially in elderly and nonelderly people [24, 25]. Thus, this set of factors may have contributed to the fact that age and the percentage of weight lost were the main independent risk factors for acute sarcopenia. On the other hand, moderate and severe cases of COVID-19 require hospitalization, and the implication for weight loss, in these cases, is the inflammatory process in response to SARS-CoV-2 [7]. Once the virus enters the cell, rapid viral replication begins, followed by a series of reactions and a storm of proinflammatory cytokines and chemokines [26, 27]. Thus, this acute respiratory syndrome results in hypercatabolism and consequent cell death, which explain the rapid weight loss that follows SARS-CoV-2 infection [28, 29]. Allard et al. [30] showed that approximately 34% of patients with COVID-19 had >5% weight loss (2.6 ± 5.9 kg) at the time of hospitalization. The mean interval between the onset of symptoms and hospitalization was 7.6 ± 5.3 days, which was similar to the duration of symptoms found in our study. The prehospitalization period of one to two weeks is a commonly investigated interval when assessing weight loss and change in intake [31]. Another study showed that 35% of patients with COVID-19 were malnourished on admission. This diagnosis was based on weight loss, that is, <5% for 66% of patients, between 5% and 10% for 23%, and >10% for 11% of cases. In that same study, the risk of sarcopenia, assessed by SARC-F, was found in 73% of patients [32]. Moreover, in addition to the respiratory condition, anorexia and gastrointestinal disorders such as dysgeusia, anosmia, diarrhea, abdominal pain, nausea, and vomiting are commonly reported symptoms that lead to low intake, malabsorption, and weight loss [33]. Our results showed that all sarcopenic patients reported anorexia upon admission in addition to other complaints such as dysgeusia, dysosmia or anosmia, nausea, and vomiting. These symptoms, although there was no statistical difference between patients with and without sarcopenia, may have contributed to the greater weight loss observed among sarcopenic patients. Still regarding the inflammatory response, our results showed no differences in PCR results between sarcopenic and nonsarcopenic patients. Thus, weight loss in patients with COVID-19 and muscle mass occur not only due to the immunoinflammatory response but also due to immobility, low intake, and digestive symptoms [11, 12]. Furthermore, our data showed that low muscle mass occurred in 22.5%, and almost half (46.5%) of patients had low muscle strength. Thus, not only the amount of muscle mass but also its quality is impaired [34–36]. Among hospitalized patients with COVID-19, Attaway et al. [37] showed a greater loss of pectoral muscle when compared to patients without COVID-19 (−2.6 vs. −0.06; *p* < 0.001). In addition, the multivariate analysis showed an association between this percentage of loss and increased mortality and ICU admission. Based on clinical observations, it is evident that people of any age with SARS-CoV-2 infection may evolve with loss of weight, muscle mass, and strength. Thus, it can be inferred that a percentage of unintentional body weight loss ≥3% and advanced age, the latter with the lowest power of association, contributed to the fact that almost 1/4 of patients with COVID-19 were already hospitalized with acute sarcopenia. Although the present investigation showed weight loss as an important factor for sarcopenia among patients admitted with COVID-19, it has limitations. First, the sample was heterogeneous in terms of sex, age (young and elderly adults), and the presence of comorbidities. Second, it was not possible to evaluate and separate patients whose sarcopenia was primary or secondary or whether there was an overlap between both, even among young adults. Third, to assess sarcopenia, the cutoff point for the elderly was used, as to date there is no consensus on the cutoff point for nonelderly patients as well as for Brazilian patients. Fourth, the use of CC for MM evaluation has inherent limitations that also should be addressed. CC assessment may be influenced by factors such as intramuscular or subcutaneous adipose tissue deposition. However, for the general elderly population, CC measurement is still a convenient method for estimating MM, and, despite its limitations, may be a useful screening anthropometric method. As an example, CC is one of the parameters used in the MNA-SF to assess nutritional status of the elderly [38]. As such, CC measures may be used as a diagnostic proxy for older adults in settings where no other MM diagnostic methods are available [10, 39]. Finally, our study points to the importance of the percentage of unintentional weight loss between the onset of symptoms and hospitalization. This loss has a negative influence on the quality and quantity of musculature in patients with COVID-19, contributing to the diagnosis of acute sarcopenia in 16% of cases.

## 5. Conclusions

Acute sarcopenia was present in 16% of patients hospitalized with COVID-19. Advanced age and ≥3% unintentional weight loss were independent risk factors for acute sarcopenia in patients hospitalized with COVID-19.

## Figures and Tables

**Table 1:** Patient demographic and clinical characteristics.

Variables	Values
Ages (M ± SD, years)	57.4 ± 15.4
Elderly (*n*; %)	92 (43.2)
Sex (*n*; %)	
Female	77 (36.2)
Male	136 (63.8)
Days of signs/symptoms of prehospitalization (M; IIQ)	9 (7–11)
Signs or symptoms of prehospitalization (*n*; %)	
Myalgia	83 (39)
Fever	118 (55.4)
Dyspnea	98 (46)
Asthenia	95 (44.6)
Anorexia	190 (89.2)
Dysgeusia/dysosmia/anosmia	71 (33.3)
Vomitus	42 (19.7)
Diarrhea	56 (26.3)
Comorbidities (*n*; %)	
Obesity	61 (28.6)
Hypertension	98 (46)
Diabetes mellitus	57 (26.8)
Body weight (M; IIQ, kg)	84 (75–98)
Usual weight (M; IIQ, kg)	86 (78–100)
Unintentional weight loss (*n*; %)	193 (90.6)
Unintentional weight loss (M; IIQ, kg)	2 (2–3)
Percentage of unintentional weight loss (M; IIQ, kg)	2.7 (2.0–4.4)
Percentage of unintentional weight loss ≥3% (*n*; %)	83 (43)
SARC-F (M; IIQ, points)	4 (2–5)
Handgrip strength (M; IIQ, kg)	30.7 (23–37.3)
Calf circumference (M ± SD, cm)	37.1 ± 4.6
Nutritional therapy (n; %)	107 (51)
PCR levels (M; IIQ, mg/L)	94 (50–145)
LOS (M; IIQ, days)	11 (5–19)
Death (*n*; %)	35 (16.4)

SARC-F: strength, assistance with walking, rise from a chair, climb stairs, fall; PCR: C-reactive protein; LOS: length of stay. Statistic presented: mean and standard deviation (M + SD), number and percentage (*n*; %), or median and interquartile range (M; IIQ).

**Table 2:** Relationship between acute sarcopenia and outcomes.

Variables	Acute sarcopenia		*p* value
	Yes (*n* = 34)	No (*n* = 179)	
Age (M ± SD, years)	67 ± 14.4	55.5 ± 14.9	<0.001^*∗*^
Body weight (M; IQR, kg)	67 (59–79)	86 (78–100)	<0.001
Usual weight (M; IQR, kg)	71 (62–84)	89 (80–102)	<0.001
Unintentional weight loss (M; IQR, kg)	3 (3–5)	2 (2–3)	<0.001
Percentage of unintentional weight loss (M; I QR)	4.8 (3.1–6)	2.5 (2.0–3.8)	<0.001
SARC-F (M; IQR, points)	5 (4–6)	4 (1–5)	<0.001
Handgrip strength (M; IQR, kg)	21 (16.2–26.2)	32 (25.3–39)	<0.001
Calf circumference (M; IQR, cm)	31.4 (29.3–32.9)	37.3 (34.8–41.5)	<0.001
PCR (M; IQR, mg/L)	92 (52–127)	94 (50–150)	0.397
Days of signs/symptoms of prehospitalization (M; IQR)	8 (6–15)	9 (7–11)	0.607
LOS (M; IQR, days)	9 (7–14)	11 (5–19)	0.943

SARC-F: strength, assistance with walking, rise from a chair, climb stairs, fall; PCR: C-reactive protein; LOS: length of stay. Statistic presented: mean and standard deviation (M + SD) or median and interquartile range (M; IQR). Statistical test performed: Student's *t*^*∗*^ or Mann–Whitney test.

**Table 3:** Association between acute sarcopenia and outcomes.

Variables	Acute sarcopenia		OR (95%CI)	*p* value
	Yes (*n* = 34)	No (*n* = 179)		
Male (%)	55.9	65.4	7 (0.32–1.41)	0.292
Elderly (%)	73.5	37.4	(2.0–10.5)	<0.001
Comorbidities (%)				
Obesity	8.8	32.4	(1.45–16.9)	0.005
Arterial hypertension	47.1	45.8	5 (0.45–1.98)	0.893
Diabetes mellitus	29.4	26.3	5 (0.38–1.92)	0.703
Signs and symptoms of prehospitalization (%)				
Myalgia	50	36.9	8 (0.28–1.2)	0.150
Fever	47.1	57	(0.71–3.1)	0.286
Dyspnea	50	45.3	2 (0.4–1.7)	0.611
Asthenia	44.1	44.7	2 (0.49–2.1)	0.951
Anorexia	100	87.2	7(0.82–0.92)	0.027
Dysgeusia/dysosmia/anosmia	29.4	34.1	4 (0.56–2.76)	0.597
Vomitus	29.4	17.9	2 (0.23–1.2)	0.121
Diarrhea	32.4	25.1	2 (0.32–1.55)	0.381
Unintentional weight loss (%)	97.1	89.4	5 (0.03–2.00)	0.160
Percentage of unintentional weight loss ≥3% (%)	81.1	35	(3.2–21.4)	<0.001
Nutritional therapy (%)	79.4	45.5	(1.9–11.2)	<0.001
Death (%)	17.6	16.2	(0.42–2.91)	0.835

OR = odds ratio; CI = confidence interval. Statistic presented: number and percentage. Statistical test performed: chi-square test.

**Table 4:** Multivariate analysis of factors associated with acute sarcopenia.

Variables	Exp (B)	95% CI	*p* value
Elderly	3.57	1.45–8.8	0.005
Percentage of unintentional weight loss ≥3%	6.93	2.65–18.1	<0.001

CI = confidence interval.

## Data Availability

The data used to support the findings of this study are included within the article.
